# Evaluation of Interceptor long-lasting insecticidal nets in eight communities in Liberia

**DOI:** 10.1186/1475-2875-9-84

**Published:** 2010-03-24

**Authors:** Kristin Banek, Albert Kilian, Richard Allan

**Affiliations:** 1 Uganda Malaria Surveillance Project, PO Box 7475, Kampala, Uganda; 2 Malaria Consortium, Development House, 56-64 Leonard Street, London EC2A 4LT, UK; 3 The MENTOR Initiative, La Prade, 11150 Villasavary, France

## Abstract

**Background:**

By 2008, the WHO Pesticide Evaluation Scheme (WHOPES) recommended five long-lasting insecticidal nets (LLINs) for the prevention of malaria: Olyset^®^, PermaNet 2.0^®^, Netprotect^®^, Duranet^® ^and Interceptor^®^. Field information is available for both Olyset^® ^and PermaNet^®^, with limited data on the newer LLINs. To address this gap, a field evaluation was carried out to determine the acceptability and durability of Interceptor^® ^LLINs.

**Methods:**

A one-year prospective field study was conducted in eight rural returnee villages in Liberia. Households were randomized to receive Interceptor^® ^LLINs or conventionally treated nets (CTNs). Primary outcomes were levels of residual alpha-cypermethrin measured by HPLC and participant utilization/acceptability of the ITNs.

**Results:**

A total of 398 nets were analysed for residual alpha-cypermethrin. The median baseline concentrations of insecticide were 175.5 mg/m2 for the Interceptor^® ^LLIN and 21.8 mg/m2 for the CTN. Chemical residue loss after a one year follow-up period was 22% and 93% respectively. Retention and utilization of nets remained high (94%) after one year, irrespective of type, while parasitaemia prevalence decreased from 29.7% at baseline to 13.6% during the follow up survey (p = < 0.001). Interview and survey data show perceived effectiveness of ITNs was just as important as other physical attributes in influencing net utilization.

**Conclusion:**

Interceptor^® ^LLINs are effective and desirable in rural communities in Liberia. Consideration for end user preferences should be incorporated into product development of all LLINs in the future, in order to achieve optimum retention and utilization.

## Background

There has been a renewed emphasis on preventive measures for malaria at community and individual levels. Long-lasting insecticidal nets (LLINs) have been strongly advocated for use to prevent malaria in sub-Saharan Africa [1,2] and are considered to be a significant improvement in the strategy to fight malaria [3]. In order for LLINs to be the most effective, the World Health Organization Global Malaria Programme has recommended that in areas were LLINs are deployed for malaria prevention, full coverage of all people at risk for malaria should be achieved [4]. However, in order to reach universal coverage, sufficient numbers of effective LLINs will need to be available; for this to happen more than one or two brands of effective LLIN products are desirable.

The BASF Chemical Company produces alpha-cypermethrin (FENDONA^®^), an insecticide recommended by WHOPES for indoor residual spraying (IRS) and treatment of mosquito nets [3]. Previous studies have shown the operational feasibility and bio-efficacy of alpha-cypermethrin when used to treat netting materials [5-7]. To meet the growing need and demand for LLINs, the BASF Chemical Company developed a LLIN in 2004. The polyester based mosquito net is treated with a long-lasting insecticidal process using the insecticide alpha-cypermethrin and a binding polymer. The product was branded Interceptor^® ^and phase I (laboratory) and phase II (small scale field) trials have shown promising results [BASF, unpublished data]. The phase I & II trials showed that Interceptor^® ^nets washed 20 times still achieved 99% mosquito mortality and inhibited blood feeding by 100%. These results were above WHO efficacy criteria [8] and subsequently the Interceptor^® ^received an interim recommendation from WHOPES [3].

By 2008, the WHO Pesticide Evaluation Scheme (WHOPES) recommended five LLINs for the prevention of malaria: Olyset^®^, PermaNet 2.0^®^, Netprotect^®^, Duranet^® ^and Interceptor^®^. Field information is available for both Olyset^® ^and PermaNet^®^, with limited data on the newer LLINs [2]. This field study was conducted in order to evaluate the performance and acceptability of using Interceptor^® ^nets under 'normal' field conditions in eight villages in Liberia.

## Methods

### Overall study design

This study was a prospective field trial with a 12-month follow-up period, based on the 2005 World Health Organization (WHO) Guidelines for Laboratory and Field Testing of LLINs [9] with slight modifications based on other field trials [10,11]. The target population included residents of eight refugee returnee villages in Liberia. Households who agreed to partake in the study were randomized to receive either a BASF LLIN or a similar conventional net treated with the same chemical (alpha-cypermethrin).

### Nets and insecticides

Interceptor^® ^nets (treated with alpha-cypermethrin using long-lasting technology) were provided by the BASF Chemical Company. The nets were white polyester material, 75 denier, rectangular in shape, 180 cms (length) × 160 cms (width) × 150 cms (height). Conventional untreated nets were also supplied by BASF. These were made of the same polyester material, shape and size as the Interceptor^® ^nets. These were treated by the study team with sachets of alpha-cypermethrin (Fendona^®^) provided by the BASF Chemical Company. The treatment of the conventional nets took place at The MENTOR Initiative Liberia premises by a trained team of malaria prevention workers. All nets were identified with a unique ID number that was written with wash resistant ink.

### Inclusion criteria and distribution

Villages that had at least 50 households were assessed for inclusion in the study. Communities that were accessible and agreed to participate were selected. All households within the selected villages who agreed to participate in the study were included. All nets were hung above sleeping areas by the study team, community health workers and household members at the time of enrolment. As the nets were hung information on how to use and care for the net was conveyed to the household members. Over the course of the intervention health education was carried out by the community health workers and committees. Education was designed to reinforce proper utilization and care of the nets, including washing, drying, the importance of not using heat sources (i.e. coal pots, candles, cigarettes, etc) under the nets and the need for repairing holes in the nets.

### Randomization

Each village was enumerated prior to the study through a household census. A complete list of households for all eight villages was generated along with a village map indicating the location of houses, environmental factors (swamps, creeks etc) as well as schools and other community areas. Randomization codes were computer-generated and then matched to the household listing for the distribution of the nets.

Based on this master listing, nets were distributed to household structures. Structures containing more than one household were treated as one group, but given separate household identification numbers for consent and data collection purposes. All households within the same structure were randomized to receive the same type of net. New structures that emerged during the distribution process due to returning population were randomized to one of the two groups. New households were given unique household identification numbers and were given the same net as the other households in that structure. Any new structures or households that appeared after the start of the study were not given nets. Participating households were informed that each household would receive either one long lasting mosquito net or one conventionally treated mosquito net. The type of net received by a household was disclosed at the conclusion of the evaluation for anyone requesting this information.

The master listing was used as the basis for selecting households for the baseline and follow-up household surveys as well as the monitoring surveys. Households were randomly selected from the master list prior to both the baseline and follow-up surveys. The methodology for the monitoring visits differed only in that net samples were taken at the time of the monitoring visit. As with the baseline and follow-up surveys, households selected for monitoring were also randomly selected from the listing. Once sampled the household was removed from the list to ensure that only households that had not been previously sampled would be sampled at each subsequent time point.

### Informed consent

All county, district, village community leaders, village committees/councils and heads of households were informed about the study prior to initiation. Village leaders and committees helped create awareness of the study within their communities. The official language of Liberia is English; all consent forms were written in "simple" English to ensure all participants understood the consent form. The head of each household was asked to sign the consent form for their household to participate in this evaluation. In some instances, this meant that more than one head of household was present in a structure. To make the consenting procedure clear to all potential participants, verbal translation in the beneficiaries' local dialect/language was provided when needed. Specific consent was received separately during the household surveys for fingertip blood samples to test for haemoglobin and parasite prevalence. Participants were informed that participation in the study was completely voluntary and that they may withdraw from the study at any time without penalty.

### Consideration of risks and benefits to the population

This field project posed a minimum risk to participants; however it was not unforeseen that residents of some households that received nets might experience some minor adverse effects (AE's) from the insecticide. AE's that have been historically associated with exposure to insecticides include skin paraesthesia, skin burning, skin redness, skin itching, eye tearing, runny nose, sneezing, watery eyes, mucosal irritation, headache and dizziness[3]. The study team carefully monitored for any potentially related AE's in the study population during supervision visits and ensured a system to report any serious AE immediately to the ethical review board.

### Household surveys

Baseline and follow-up household surveys were conducted prior to net distribution and after 12 months of use. Information was collected on net ownership, utilization, history of fever or illness as well as blood samples to determine anaemia (Using Hb 201+ machine; HemoCue AB Ängelholm Sweden) and parasitaemia (via RDT; Paracheck Orchid Biomedical, Goa, India) in children less than 5 years of age. Any participants found to be anaemic or testing positive for malaria during these screenings were treated with ferrous sulphate and/or ACT.

### Monitoring surveys

Households were followed and monitored by the study team at months 1, 3, 6, 9 and 12. During the monitoring visits information on utilization, net condition and perceived side effects of the nets were collected. Additionally, samples of ITNs were also collected and sent for testing of residual insecticide.

### Qualitative data collection

Focus group discussions (FGD) were carried out at months 3, 9 and 12. A list of standardized questions was developed to guide the discussions and included all types of ITNs available in the communities. Each FGD had a discussion leader and a recorder. Four FGD were carried out in each of the eight villages: men, women, youth and elders/leaders. Key Informant Interviews (KII) were also conducted with influential community members who were selected for one-on-one or small group interviews. Participants were either interviewed collectively as per category or individually; whichever was most appropriate and convenient so long as they were uninfluenced and independent responses were guaranteed.

### Chemical residue procedures

Net samples were analysed at the Overseas Merchandise Inspection Co., Ltd Laboratory in Bangkok, Thailand by High Performance Liquid Chromatography (HPLC) using the Collaborative International Pesticides Analytical Council (CIPAC) protocol for extracting alpha-cypermethrin (unpublished protocol received by personal communication). The laboratory test results recorded the insecticide concentrations for each sample in g/kg. This was converted to the standard measurement of mg/m^2 ^for the analysis. As the surface area was not known for each individual sample (four samples per net) the calculation was carried out using the WHO Standard of 30 g/m^2 ^for the mass per surface area for a 75 denier polyester net [12].

### Statistics and analysis

#### Sample size calculations

The sample size calculation for the monitoring surveys was based on the estimated percentage of children less than five years of age sleeping under a mosquito net the previous night (8%) [13], thus yielding a total of 84 households. The monitoring survey corresponded with the collection of net samples (42 LLINs and 42 CTNs) for laboratory testing. Based on the WHO guidelines, a total sample of 30 nets collected every six months is adequate to detect changes in performance (washing durability and efficacy) over time [9].

#### Data collection and quality

All members of the study team were educated in the study protocol prior to the onset of the study. The study team completed data forms as indicated in the monitoring schedule. These forms were reviewed by the study coordinators for completeness and accuracy. Field team meetings were conducted monthly by the coordinator to collect data forms, assess progress of the study, address any difficulties, and provide performance feedback to the members of the field teams.

All data was transferred from the data forms into a computerized database (EPI INFO 6.04 and ACCESS) by data entry personnel. Data was double entered to verify accuracy of data entry whenever possible. Back-up files of the database were created on a weekly basis. For quality control, check programmes were written into the database to limit the entry of incorrect data and to ensure entry of data into required fields.

#### Analytical plan

Data analysis was performed by the project team using EpiInfo6 and STATA statistical software packages. Descriptive statistics were used to summarize baseline characteristics of participants. Categorical variables were compared between the two groups using chi-square tests or Fisher's exact tests and continuous variables were compared using t-tests. A p-value of < 0.05 was considered significant.

#### Ethical approval

This study was approved by the Ministry of Health and Social Welfare, the National Malaria Control Programme and the Liberian Institutional Review Board.

## Results

### Household characteristics

1,710 households were approached to take part in the study. A total of 1,688 households received nets as per randomization. An additional 22 households were given nets, but were not randomized and were, therefore, excluded from the follow-up cohort. 910 households received the BASF Interceptor^® ^LLIN and 778 received the conventionally treated net (CTN). Of the households randomized to receive nets, 1,299 were eligible to take part in the study (signed informed consent and no associated protocol violations). Households were considered a protocol violation if they did not provide informed consent, received a net but were not randomized, received the wrong net or were sampled more than once during monitoring visits.

### Monitoring data

After correcting for the ten nets in storage and those associated with protocol violations or missing data, 384 nets were eligible for the monitoring survey analysis; 197 LLINs and 187 CTNs. During the monitoring visits, the majority of nets (98.4%) were found to be hanging over sleeping places, 1.3% were stored or folded away and one net (0.3%) was hung up in the shade to dry after washing at the time the monitoring survey was conducted. Information on how the net was hanging over the sleeping space (i.e. folded up or hanging down) was not collected.

### Net care

Out of the 384 nets that were eligible, 275 (71.6%) were reported to have been washed at least once during the 12 month follow up period, with an average number of two washes (95% CI 1.8 - 2.1). The maximum number of times a net had been washed was reported to be 14. The average number of days since the last wash at the time of monitoring was 30 days (95% CI 20.6 - 40.8). Of those reported to have washed their nets, 98.9% used cold or normal water. Only two respondents reported using warm or hot water. Four types of soap were used to wash the study nets; 60% of nets were washed using locally made soap, 19% were washed using detergent such as the bathroom cleaner, such as "vim", 10% of nets were washed using laundry soap, such as "omo', and 11% were washed using bath soap. Nets were reported to be dried in a hanging position the majority of the time (96.7%). Most nets (77%) were dried in the shade while 22% were dried in the sun similar to other household laundry. A very small percentage (1%) reported spreading their nets out on their beds to dry.

### Adverse effects

No severe adverse effects were reported specifically for either the Interceptor^® ^LLIN or the conventionally treated net. A total of 68 households (17.7%; 95% CI 13.9-21.5) reported having experienced a range of minor adverse effects ('unusual feeling') while using the study nets. The incidence of reported minor adverse effects was similar for those households with Interceptor^® ^and those with conventional treated nets. Most adverse effects (91.2%) were reported within the first three months of the project. During the focus group and key informant interviews the time frame was found to be narrow and focused around the month directly following the distribution of the nets.

Adverse effects such as burning (45.6%) and itching (41.2%) were reported as the most common experienced. Other adverse events reported include feeling "heat/hot" or "warm" (4.4%), headache, runny nose or cold (1.5%) and "running stomach"/diarrhoea (2.9%). Of the minor adverse effects reported, 90.7% lasted one week or less, with the majority lasting just 1-3 days (77.3%). Three persons reported that the adverse reaction lasted 2-3 weeks. Four persons didn't specify the duration.

### Physical condition

**Figure 1:**
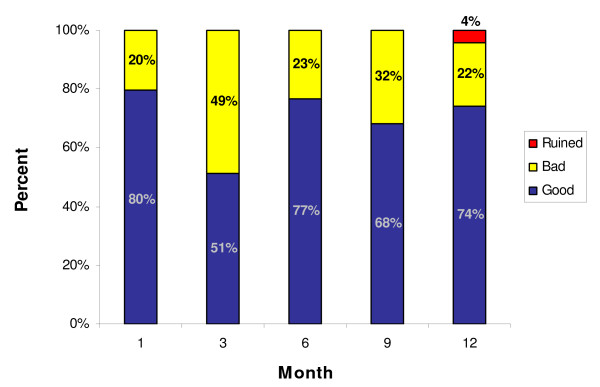
**Net condition by month**.

**Figure 2:**
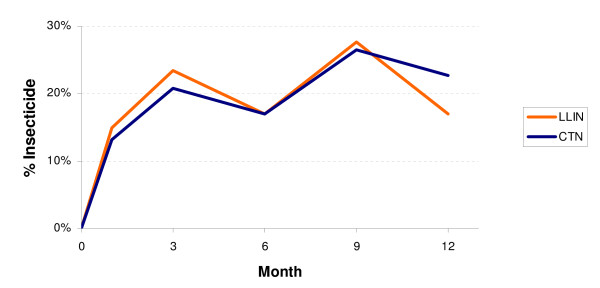
**Proportion of nets with holes by month**.

Of the 384 nets monitored, inspection data was available for 383 nets. 70% of the nets inspected were found to be in good condition, i.e. clean with no holes. Over the course of the 12-month follow-up period an average of 29% (range 10-49%) of nets were found to be in bad condition, (i.e. dirty/not maintained and/or with one or more holes). After one year of use, only three nets were classified as 'Ruined' (classified as having too many holes to be repaired) (Figure 1). The average number of holes per net was 2.5, with a minimum of one hole and a maximum of 17. The overall proportion of study nets with one or more holes over the study period was 26.6% (100/383). The proportion of Interceptor^® ^LLINs with one or more holes was 47/196 (24%) compared to the conventionally treated nets of 53/188 (28%) is illustrated in Figure 2. The difference in proportion of nets with holes was not statistically significant.

### Chemical residue

**Figure 3:**
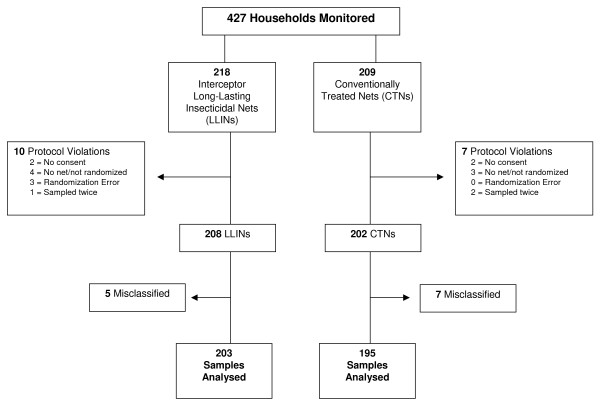
**Study profile for residual chemical testing**.

A total of 427 household sample sets were collected for chemical testing (Figure 3). Due to protocol violations and misclassification of some results 29 samples were dropped from the analysis. The total number used in the analysis was 398 samples (203 Interceptor^® ^LLINs and 195 conventional nets).

Tables 1 and 2 display the mean alpha-cypermethrin concentration at baseline for the Interceptor^® ^LLIN (180.1 mg/m^2 ^[95% CI 152.5-207.7]) compared to the conventional treated net (21.2 mg/m^2 ^[95% CI 15.8-26.5]). The median alpha-cypermethrin concentration at baseline was 175.5 mg/m^2 ^for Interceptor^® ^LLIN and 21.8 mg/m^2 ^for the conventional net. After 12 months, the concentration of alpha-cypermethrin in the Interceptor^® ^LLIN was still quite high, with a mean 126.2 mg/m^2 ^(95% CI 113.1 - 139.4) and median of 137.9 mg/m^2^, and the conventional dipped ITN had a mean of 4.8 mg/m^2 ^(95% CI 2.9 - 6.7) and median of 1.5 mg/m^2 ^.

**Table 1:** Concentration of alpha-cypermethrin by net type: Interceptor LLIN

Month	n	Mean mg/m^2^	95% CI	SD	Median mg/m^2^	Range	IQR
0	5	180.1	152.5 - 207.7	22.3	175.5	53.3	31.2
1	41	178.5	167.6 - 187.3	28.2	183.2	100.7	36.7
3	37	167.8	156.8 - 178.8	33.0	161.8	137.9	48.9
6	45	140.5	129.5 - 151.5	36.6	137.6	197.5	35.0
9	38	148.5	135.6 - 161.5	39.4	162.0	162.0	49.4
12	37	126.2	113.1 - 139.4	39.4	137.9	137.9	58.7

**Table 2:** Concentration of alpha-cypermethrin: Conventionally treated net

Month	n	Mean mg/m^2^	95% CI	SD	Median mg/m^2^	Range	IQR
0	5	21.2	15.8 - 26.5	4.3	21.8	11.6	1.4
1	39	16.5	13.8 - 19.3	8.4	18.1	41.4	14.2
3	41	12.4	8.4 - 16.3	13.5	7.5	79.0	14.2
6	34	5.8	3.5 - 8.0	6.4	2.3	20.1	4.6
9	38	7.4	3.4 - 11.4	12.2	1.5	66.5	11.3
12	38	4.8	2.9 - 6.7	5.8	1.5	17.3	2.9

**Figure 4:**
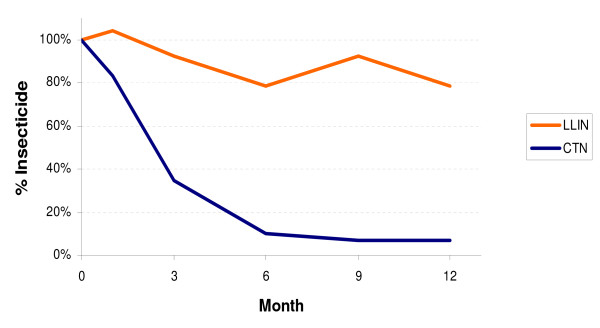
**Median proportion of insecticide from baseline**.

The mean percent loss of alpha-cypermethrin after 12 months compared to baseline for the Interceptor^® ^LLIN is 30% and the conventional net 80%. The median percent loss of alpha-cypermethrin for the Interceptor^® ^LLIN over the follow up period was 22% compared to a 93% loss for the conventionally treated net (Figure 4).

Table 3 presents the between and within net variation was calculated for each net at each time point. Between net variations for the Interceptor^® ^gradually increased over time, but remained close to the WHO standard of 25%. However, the variation between conventional nets increased almost six-fold over the follow-up period.

**Table 3:** Between and within net variation

	Interceptor		Conventional	
**Month**	**Between net variation**	**Within net variation** **(4 samples per net)**	**Between net variation**	**Within net variation (4 samples per net)**
	Coefficient of variance*	**Avg. difference to mean in % of mean**	Coefficient of variance*	**Avg. difference to mean in % of mean**

0	12.2%	7.2%	20.5%	16.3%
1	15.8%	7.5%	51.0%	19.5%
3	19.6%	8.2%	109.4%	19.7%
6	26.0%	9.6%	112.1%	15.8%
9	26.5%	9.0%	165.6%	15.9%
12	31.2%	11.5%	119.2%	11.9%

*Standard Deviation expressed as % of mean

Within-net variation (difference between the four samples per net) for the Interceptor^® ^LLIN increased slightly, but still remained relatively the same over the follow-up period. Equally, the conventional net within net variation was also minimal, with variability decreasing over the follow-up period.

### Household survey data

Results from the household survey show that overall household ownership increased from 11% to 97%. Data from the household surveys illustrated that utilization of nets in those households owning nets also improved, with the proportion of households reporting 'anyone sleeping under the mosquito net the previous night' increasing from just 11% at baseline to 94% at the end of the intervention; the proportion of persons protected by sleeping under a mosquito net the previous night was found to be 3% (44/1,574) at baseline and 55% (729/1,332) persons at follow-up. The proportion of children under five years of age sleeping under the net the previous night was 65% (242/371) and the proportion of pregnant women was 52% (17/33).

The average number of persons sleeping under the net the previous night was the same for both surveys (2.4 persons/net). The survey tool limited the maximum number of persons under a net to four with the opinion that more than four persons per net would be a rare event. However, through direct observation and discussions with the community, it seems likely that in some cases the ratio of people sleeping under a net exceeded 4:1. A single household may accommodate an extended family with each unit of the extended family occupying one room in the house. The rooms varied in size; however they were often only large enough to hold one bed, which all the room occupants shared.

### Impact

The proportion of positive rapid diagnostic tests decrease from 29.7% at baseline to 13.6% during the follow up survey (p = < 0.001). The number of reported fevers actually increased from 16.5% at baseline to 20.7% at the end of the intervention. However, this change was not significantly different (p = 0.17). The proportion of reported fever cases who were actually RDT positive declined from 47% at baseline to only 11% of reported fever cases having positive RDTs after the intervention (p = < 0.001, Fishers exact test).

The proportion of children with clinical malaria, defined as positive RDT plus fever, out of all children tested for each of the surveys was used to determine the point prevalence of clinical malaria. At baseline 7.7% (95% CI 5.1 - 11.1) of children tested had clinical malaria compared to 2.2% (95% CI 0.9 - 4.2) of children tested during the follow-up survey with an odds ratio of 0.27 (p = 0.0006).

Mean haemoglobin at baseline was 9.8 g/dl (minimum 3.3 and maximum 17.5) and 10.1 g/dL (minimum 4.2 and maximum 17.7) during the follow-up survey. Median haemoglobin at baseline and follow-up were 10.2 g/dl and 10.3 g/dl respectively. Measurements were also categorized into moderate/severe anaemia (< 8 g/dl) and moderate/normal anaemia (> 8 g/dl). Results from the categorization show that 21 (6.6%) children had moderate/severe anaemia at baseline compared to 29 (9.1%) during the final survey. This difference in moderate/normal anaemia was not found to be significantly different (OR = 0.70; p = 0.2341).

### Qualitative results

#### Acceptability

Nets distributed by the Ministry of Health were preferred by some respondents due to its size. However, they doubted its ability to protect them against mosquitoes, given the large mesh size of this net. Due to its texture some people also liked to use it as a fishing net or "saphoon" a local bathing sponge. In reference to these polyethylene nets, some respondents said: *"I do not prefer this net because it is very hard and rough" *or *"... it has big big holes that mosquitoes may pass through."*

When asked about physical differences between all four types of nets available in the community (Study nets, other polyester nets, and polyethylene nets) only the polyethylene net was singled out as being 'different'. Most respondents were confused when it came to making a preference among the study LLIN, CTN and other polyester nets available due to the similarity in texture and design of the nets. Given that few other polyester nets were found in the villages, in most instances the respondents would refer to and speak about their study nets when discussing which net they preferred. For example, many respondents pointed out the study net as their net of preference because, according to them: *"This one (study net), it get medicine on it. It has been helpful to us. In fact, this net that was given us [study nets], this, is far yonder better than these people (the Hospital) give us.... I have observed that it [study net] has medicine in it and it kills flies and other insects."*

This physical evidence of killing mosquitoes, flies and other insects was found to be an important factor for acceptability and thus utilization of the ITNs. Some respondents admitted their intention to take the net to their farms whenever they had to sleep there for a night or two, further demonstrating their belief that the study nets were effective and beneficial. During the interviews it became apparent that any noticeable differences between the CTN and the interceptor LLIN went unnoticed by respondents as the nets were very similar in terms of texture and size and initial 'killing action.' The biggest difference noted were the slight variation in hanging hardware: *"The type of net I have is the one that has a ring on it" *or *"Mine is the type that has a rope handle with writing." *Although some participants reported "*itching and burning sensation" *during the initial utilization of either net [*"First time they hung it, it itched my skin*], no serious adverse effect was attributed to a specific net.

#### Design preferences

A number of respondents expressed interest in having a larger variety of net colours available since whites nets, according to them *"are hard to maintain [keep clean]." *Similar findings were found during the household surveys that show that approximately 50% of respondents interviewed preferred blue nets. Despite this preference, the white study nets were still utilized as a result of both understanding as well as witnessing for themselves the initial killing action and benefits of mosquito nets, irrespective of colour.

Some respondents also expressed preference for bigger nets and preferred the polyethylene nets distributed by the ministry of health for its size. However, other respondents reported that their rooms were very small and that they were satisfied with the size of nets they had. A young man living in Sappimah Town said in reference to the Interceptor^® ^size: *"My bed as you can see is so big that the net won't cover. I am just managing somehow. I will be happy if you people will bring bigger nets."*

There were also views about the shape of nets. Some respondents recounted the "*inconvenience*" in tying the nets to various points on their mat ceilings and are under the impression that a ring or circular shape net that has one fastener or hook might be more preferable.

#### Willingness to pay

In an effort to establish study participants' regard for ITNs, respondents were asked as to what they would do should there be no more free nets. Although some wished that the distribution of free mosquito nets would continue, other responses were surprising, stating: *"We will seek to purchase nets for ourselves." *When asked how much, they would be willing to spend for a mosquito net, responses ranged from L$ 200.00 to L$ 600.00 (i.e. US$ 4.00 to US$10.00) per net. However, the majority said that they would pay around L$300.00 (US$5.00) per net. A school Teacher in Guyanta, referring to the Interceptor^® ^ITN, said: *"I can pay as much as four hundred [Liberian] dollar or even 450 [Liberian dollar] for a net of this quality"*

Chief Farwein of Farwenta Town demonstrated not only his willingness to pay for nets, but also his understanding of why they are so important. *"Now that we know how much helpful the mosquito net is to us.... Me, so long as it pertains to my health, I can pay whatever possible, even 600 [Liberian dollars] to protect myself from sickness. I will harvest and sell my crops to get it. I will buy it for my protection. ....For my protection, even if they tell me 100 or 200 [Liberian] dollars I will buy because I am buying it for my own protection. It's better I spend [the money] and get well than to be sick at all times."*

## Discussion

The overall proportion of ITNs, which retained good material condition during the course of this study, was 97%. Despite expected human factors that can have had an effect on ITN physical conditions, no significant differences were observed between the material integrity of the LLIN versus the CTN.

Despite similarities in material strength, the Interceptor^® ^LLINs significantly out performed CTNs in retaining insecticide over time. At 12 months Interceptor^® ^contained an average of 126 mg/m^2 ^of residual insecticide, which is above the level normally associated with effective vector control when using alpha-cypermethrin (40 mg/m^2^)[14]. Although we can not say from our data that the insecticide was also available on the surface of the net this is very plausible based on the findings presented to WHOPES [3], whereby the Interceptor^® ^was found to maintain biologically activity for at least 20 washes under field conditions as per WHO guidelines [9]. Future field-testing of the Interceptor^® ^LLIN should include bio-assay testing in order to capture more accurate bio-efficacy data under field conditions.

Although the conventional net was on the lower end of the WHO recommended target dose of 20-40 mg/m^2 ^from the onset, concentrations were consistent with other studies where alpha-cypermethrin was used to manually dip conventional nets [6,7,15]. Chemical residue loss was rapid and by the third month of the study the conventional net was already below 25% of the WHO recommended concentration for nets [14] and by 12 months had lost all but 1.6 mg/m^2 ^of insecticide. This was expected as insecticide when applied conventionally to nets, simply coats the textile fibres and is not 'bound' in a durable manner to the net leading to a reduction in residual insecticide levels due to washing frequency or with time [3]. However other factors such as washing technique, frequency or exposure to sunlight should not be discounted.

Although shape, texture and colour all contribute to the overall acceptability of ITNs in these communities in Liberia, the strongest preference expressed during interviews with households was the physical evidence that a net was treated with insecticide. This was demonstrated by people witnessing dead mosquitoes and insects. Study households reported a noticeable reduction in the presence of mosquitoes in their homes; they also cited reduction of other insects (cockroaches, flies, etc). Despite the community's ability to differentiate between the Interceptor^® ^LLIN and the conventionally treated nets, the initial impression of effectiveness was found to be a key determinant to acceptability, as utilization remained high despite the sharp decrease in chemical residue in the conventionally treated nets over time.

Acceptability of nets treated with alpha-cypermethrin such as the Interceptor^® ^is very high in Liberia. Preference was shown by households for polyester nets (equally for Interceptor^®^, conventionally treated nets and other available polyester) over polyethylene nets, due to their softer textures. Additionally, larger mesh size is perceived to be a negative attribute as mosquitoes can enter. Furthermore the larger mesh size was found to be suitable for other non-intended purposes such as fishing and bathing aids. Most importantly the perceived effectiveness of the ITN (i.e. physically witnessing dead insects) appears to be the determining factor as to whether an ITN will be correctly used. Respondents did not believe ITNs were treated if they did not see the dead insects and therefore did not believe it would protect them or their families from malaria.

Community members liked the study nets (differences between the two nets were unknown) so much that they reported that they would even pay for nets if free nets were no longer available in Liberia. Amounts ranging from 200-450 Liberia Dollars (~$4-7 USD) were mentioned. In a country where social services country-wide is still far below pre-war levels and more than one third of the population, and an even higher proportion of the country's children, lives on less than $1 a day[16], this finding affirms the high priority and acceptability that these poor rural Liberian communities have for nets.

The prevalence of clinical malaria (measured by point prevalence) by the end of the 12-month study period reduced significantly (p = 0.0006) in children less than five in the overall study population. Although the residual insecticidal activity of conventional nets reduced sharply over time and the Interceptor^® ^maintained sufficient insecticide thought to be effective in field conditions, the basis for this reduction cannot be singled out as the result of using the Interceptor^® ^LLIN.

In parallel with this field study, the National Malaria Control Programme and partners in Liberia were improving access to artemesinin combination therapy (ACT), the national first line therapy for uncomplicated malaria at health facilities throughout the country. Although, only one study village actually had a clinic within its boundaries, the remaining seven study villages had access to free and effective anti-malarial treatment. The direct impact of the increased availability of ACT at health facility level in this area has not been measured or accounted for in this study, however access to ACT for uncomplicated malaria treatment has been shown to reduce incidence of severe anaemia [17] and clinical disease. However, more comprehensive and integrated approaches which address both treatment and prevention aspects of the disease are now encouraged [18,19].

Although the proportion of positive RDT tests reduced over the period of the study, the number of fever cases remained relatively unchanged. This study suggests that fever alone is a non-specific disease symptom and is not a definitive indicator for malaria in this region. This finding confirms other recently published studies [20] that fever is an unreliable clinical indicator for malaria and reinforces the importance of maintaining effective confirmatory malaria diagnosis, prior to treatment of suspected malaria cases of all ages. As malaria control programmes scale up coverage of both effective treatment and prevention interventions, it will be even more essential that accurate and easy to use diagnostic tools be included within the malaria control package.

Although low haemoglobin levels in children under the age of five years old have been recommended as an effective indicator for malaria control in endemic areas [21] this does not appear to hold true in these Liberian returnee communities. The lack of significant changes in haemoglobin levels among children less than five years of age in this study is likely due to the high level of access to effective anti-malaria treatment and possibly attributed to local diet, which includes meat from forest animals. As a result the syndrome of poorly treated chronic malaria normally associated with low HB in other studies [22-28] may be negligible in this area.

Large-scale ITN distribution campaigns have been conducted in many countries in Africa since 2002 as part of Global Fund, UNICEF, IFRC, NGO and the Presidential Malaria Initiative (PMI) programmes. The distribution methodology used for these campaigns have varied, with few offering assistance to hang the ITNs. This study was not designed to test the distribution or education methodology; however it did show potential benefits of actively involving the community leadership (chiefs, elders and community health workers) in the implementation of malaria prevention activities as utilization was high. Future LLIN acceptability and durability studies should also take into account community involvement (i.e. physically hanging the nets at the time of distribution and continuous monitoring of mosquito nets post distribution) and its potential effect on utilization and retention rates. The results of this study show, that it is also important to document the perceived effectiveness and durability and not only the actual measured effectiveness and durability of the LLIN; perception of advantages could be a bigger determinant of utilization and retention rather than the actual measured benefit.

## Conclusions

This field study demonstrates that nets treated with alpha-cypermethrin (both conventional and long-life) are well accepted. Interceptor^® ^LLINs are shown not only to be desirable, but also effective in contributing to reducing the burden of malaria when used correctly and supported with targeted information, education and communication in rural communities in Liberia. Challenges to acceptability and utilization of alpha-cypermethrin nets are similar to those of deltamethrin nets and are centred on the consumers' personal preferences and behaviours. Consideration for these end user preferences should be highlighted during product development, in order to achieved optimum retention and utilization.

## Competing interests

The authors declare that they have no competing interests.

## Authors' contributions

KB assisted with the design of the study, was responsible for the implementation and supervision of the study, analysed and interpreted the data and drafted the manuscript. AK participated in the analysis and interpretation of data as well as writing and editing the manuscript. RA designed the study and contributed to and edited the manuscript. All authors read and approved the final manuscript.
